# Predicting the accumulation of storage compounds by *Rhodococcus jostii* RHA1 in the feast-famine growth cycles using genome-scale flux balance analysis

**DOI:** 10.1371/journal.pone.0191835

**Published:** 2018-03-01

**Authors:** Mohammad Tajparast, Dominic Frigon

**Affiliations:** Microbial Community Engineering Laboratory, Department of Civil Engineering and Applied Mechanics, McGill University, Montreal, Quebec, Canada; Universite Paris-Sud, FRANCE

## Abstract

Feast-famine cycles in biological wastewater resource recovery systems select for bacterial species that accumulate intracellular storage compounds such as poly-β-hydroxybutyrate (PHB), glycogen, and triacylglycerols (TAG). These species survive better the famine phase and resume rapid substrate uptake at the beginning of the feast phase faster than microorganisms unable to accumulate storage. However, ecophysiological conditions favouring the accumulation of either storage compounds remain to be clarified, and predictive capabilities need to be developed to eventually rationally design reactors producing these compounds. Using a genome-scale metabolic modelling approach, the storage metabolism of *Rhodococcus jostii* RHA1 was investigated for steady-state feast-famine cycles on glucose and acetate as the sole carbon sources. *R*. *jostii* RHA1 is capable of accumulating the three storage compounds (PHB, TAG, and glycogen) simultaneously. According to the experimental observations, when glucose was the substrate, feast phase chemical oxygen demand (COD) accumulation was similar for the three storage compounds; when acetate was the substrate, however, PHB accumulation was 3 times higher than TAG accumulation and essentially no glycogen was accumulated. These results were simulated using the genome-scale metabolic model of *R*. *jostii* RHA1 (*i*MT1174) by means of flux balance analysis (FBA) to determine the objective functions capable of predicting these behaviours. Maximization of the growth rate was set as the main objective function, while minimization of total reaction fluxes and minimization of metabolic adjustment (*environmental* MOMA) were considered as the sub-objective functions. The *environmental* MOMA sub-objective performed better than the minimization of total reaction fluxes sub-objective function at predicting the mixture of storage compounds accumulated. Additional experiments with ^13^C-labelled bicarbonate (HCO_3_^−^) found that the fluxes through the central metabolism reactions during the feast phases were similar to the ones during the famine phases on acetate due to similarity in the carbon sources in the feast and famine phases (i.e., acetate and poly-β-hydroxybutyrate, respectively); this suggests that the *environmental* MOMA sub-objective function could be used to analyze successive environmental conditions such as the feast and famine cycles while the metabolically similar carbon sources are taken up by microorganisms.

## Introduction

Biological wastewater resource recovery systems are often highly dynamic with regard to availability of external electron donors [1]. Thus, the biomass is subjected to cycles of feast and famine, with the feast phase being the period when external electron donors (i.e., carbon and energy source in the case of heterotrophic growth) is available, and the famine phase being the starvation period when growth and maintenance energy is from the oxidation of internal storage compounds or of functional macromolecules [1]. Such cycles profoundly affect the microbial ecology and the metabolism of bacterial species present in these environments. One of the main metabolisms found under this cycling is the accumulation (during feast) and consumption (during famine) of storage compounds such as poly-β-hydroxybutyrate (PHB), glycogen, and triacylglycerols (TAG) [2]. Note that nutrient availability does not limit biomass formation during feast phase, but the anabolic machinery (especially the protein synthesis system) is not fully induced, which cause the biomass formation to be lower than what could be possible by the near maximum specific substrate uptake; the substrate taken-up above the rate of biomass formation is directed to the formation of storage compounds [3]. In addition, bacterial species accumulating storage compounds during the feast phase outperform the species lacking this ability probably by maintaining the capacity to rapidly respond to substrate arrival such that the metabolic cost of storage in terms of biomass yield is lower than the cost of maintaining metabolic functions to rapidly gain access to the substrate and win ecological competition [3]. Therefore, understanding and properly modeling the metabolism of the storage compounds is central to predict the population dynamics in environments such as activated sludge wastewater resource recovery systems. Once developed, these predictive capabilities will facilitate optimization of reactors designed to produce value-added by-products from wastewater.

Genome-scale metabolic models are mathematical representations of the stoichiometry of the biochemical networks based on genomic information. It is one of the tools to make the metabolic predictions on the behaviour of microorganisms in different environmental conditions. Predicting the composition mixture under various growth conditions can be accomplished by solving the genome-scale metabolic model at steady-state using flux balance analysis (FBA). This approach typically yields a set of underdetermined mass balance equations. The solution space of this equation system can be investigated by finding solutions optimally satisfying biologically relevant linear objective functions implemented in a linear-programming approach [4, 5]. Defining relevant objective functions for metabolic predictions in the environmental conditions remains at its infancy; for instance, few biologically-appropriate constraints have been imposed on optimal metabolic fluxes predicted by FBA in order to better simulate phenotypic behaviours of microorganisms at different environmental and physiological conditions such as intracellular crowding [6], constrained allocation flux balance analysis [7]. A wide-scope review on different constraint-based reconstruction and analysis (COBRA) methods on metabolic genotype-phenotype relationship was published elsewhere [8]. Therefore, the goal of the current work is to evaluate the performance of some of the objective functions available in the literature for their capacity at predicting the mixture of storage compounds accumulated by *Rhodococcus jostii* RHA1 under feast-famine cycles.

*R*. *jostii* RHA1 was isolated from lindane contaminated soil [9] and is best known for its superior ability to degrade a wide variety of organic compounds such as polychlorinated biphenyls (PCBs). The genome of *R*. *jostii* RHA1 has already been sequenced and annotated [10]. Its genome comprises of 9.7 Mbp arranged in four linear replicons: one chromosome and three plasmids [10]. Its genome encodes a surprisingly large number of oxidoreductases, enzymes that are often involved in the hydroxylation and cleavage of aromatic compounds. This is in line with the degradation capability of a wide range of xenobiotic compounds, sterols and steroids of *R*. *jostii* RHA1 [11, 12].

Two sub-objective functions to predict the fluxes of three storage compounds through *R*. *jostii* RHA1’s metabolism under feast-famine cycles were evaluated in the current study. First, following evolutionary principles, the maximal cellular growth rate/yield (both definitions are the same because carbon excretion was not allowed) was used as the principal objective function as this would likely represent the winning phenotype in a given niche [13]. Then, the two different sub-objective functions were coupled with the principal objective function to represent other metabolic/environmental constraints. Defining a first sub-objective function, it is possible that organisms pursue a global optimal use of their metabolic network independently in each growth phase (i.e., independently during the feast and famine phases) by minimizing the total metabolic fluxes (i.e., maximizing cellular efficiency) [14]. Another possible sub-objective function could be to minimize the flux differences between feast and famine growth, which would minimize the metabolic cost and time of cellular rearrangements between growth phases. In other words, although the metabolic network (e.g., minimum of total fluxes) could vary with electron donor used in the feast and famine phases (e.g., glucose and PHA, respectively), it was hypothesized that breaking down protein-enzymes and expressing new ones to reach the two optima require a high energetic investment, and lead to a significant time lag between phases. Instead, the enzymatic make-up of the cell would reach an average of the expression in both phases, and ultimately lead to similar metabolic fluxes. This sub-objective function was first developed for the analysis of gene mutations under the name minimization of metabolic adjustment (MOMA) [15]. Thus, we called it *environmental* MOMA [16].

In the current study, the storage metabolism of *Rhodococcus jostii* RHA1 during the feast-famine growth cycle was studied and modeled by FBA using its recently published genome-scale metabolic model: *i*MT1174. The genus *Rhodococcus* is important for both environmental and biotechnological applications because of its extraordinary capacity for metabolizing recalcitrant organic compounds [12]. Several representatives of this genus have been isolated from soil, marine environment and activated sludge wastewater resource recovery systems [17], making it an interesting model heterotroph to study. Like other microorganisms in environments where transient nutrient-limitation is common, *Rhodococcus* spp. can accumulate storage compounds for subsequent utilization by cells as endogenous carbon sources and electron donors during periods of nutritional scarcity [18]. Of special interest for this work, *R*. *jostii* RHA1 is able to simultaneously accumulate a variety of carbon storage compounds: poly-β-hydroxybutyrate (PHB), poly(3-hydroxyvalerate) (PHV), triacylglycerols (TAG), and glycogen [16, 18]. However, the composition of the storage compounds mixture quantitatively changes with substrates, providing an interesting case to evaluate modeling approaches.

The experiments consisted in growing *R*. *jostii* RHA1 in semi-batch conditions leading to feast-famine cycles in mineral salt medium containing either glucose or acetate as the sole carbon source. The fluxes through the glycogen, PHB, and TAG storage compounds were determined, and the genome-scale metabolic model *i*MT1174 [16] was solved with the objective functions to predict the measured storage fluxes. As we propose a new application of the MOMA principle, the fluxes of the central metabolic reactions of *R*. *jostii* RHA1 were determined during the feast and famine cycle by labelling amino acids with ^13^C-bicarbonate added at the beginning of either the feast or the famine phases. The labelling patterns were then simulated using ^13^C-metabolic flux analysis (^13^C-MFA).

## Materials and methods

### Bacterial strain and growth medium

For all reactor experiments, *R*. *jostii* strain RHA1 was grown on a mineral salt medium (MSM) containing NH_4_Cl (84.00 mg-N/L), KH_2_PO_4_ (224.54 mg/L), MgSO_4_.7H_2_O (273.59 mg/L), KCl (111.83 mg/L), and trace element solution (1 mL/L) [19, 20].The trace element solution contained: ethylenediamine tetra-acetic acid (EDTA: C_10_H_16_N_2_O_8_) (50.00 g/L), ZnSO_4_.7H_2_O (22.00 g/L), CaCl_2_ (5.54 g/L), MnCl_2_.4H_2_O (5.06 g/L), FeSO_4_.7H_2_O (4.99 g/L), (NH_4_)_6_Mo_7_O_24_.4H_2_O (1.10 g/L), CuSO_4_.5H_2_O (1.57 g/L), CoCl_2_.6H_2_O (1.61 g/L) dissolved in 1000 mL dilution water; adjusted to pH 6.0 with KOH [20]. Glucose or acetate was added to the medium as the sole carbon and energy source to final concentrations of 1.89 and 1.86 g/L, respectively. These concentrations are equivalent to 504 mg-COD/L [19]. It should be noted that C/N ratio was 9.0 and 8.8 g-C/g-N on glucose and acetate, respectively, in order not to limit microbial growth.

The purity of the *R*. *jostii* RHA1 culture during reactor cultivation was ascertain by serial dilution plating on Nutrient Agar (Fisher Scientific, Ottawa, Canada); and the identity and genetic stability of the culture was determined by plating them on MSM agar (1.5% w/v) plate supplemented with biphenyl (few crystals on the lid of the Petri dishes) [21]. All plate incubations were at 28°C.

### Reactor condition

A 2-L (working volume) sequencing batch reactor (SBR, Labfors reactor, INFORS HT, Switzerland) without settling was employed to grow *R*. *jostii* RHA1 in dynamic conditions leading to feast and famine cycles. The hydraulic residence time (HRT) and the solids residence time (SRT) were both 24 h. Continuous operation of the SBR was based on a 6-h cycle comprising 5 phases: (1) 11-min aeration and mixing start period, (2) 2-min addition of dilution water (464 mL), (3) 4-min addition of 14× stock MSM (36 mL), (4) 338-min reaction phase, and (5) 5-min effluent withdrawal (500 mL). The cycle length was set to a length similar to an activated sludge wastewater treatment systems. A few drops of anti-foam (anti-foam 204, Sigma-Aldrich, St. Louis, MO) were also added to the reactor at each cycle to prevent foaming. The inlet air flow rate was controlled with a mass flow controller to 1.5 L/min, and stirrer speed was maintained at 300 rpm. The pH was controlled at 7.0 by automatically titrating 1 M KOH or 1 M HCl solutions. The temperature was fixed at 28°C using a water jacket.

The steady-state operation of the SBR was achieved when for a few days the length of the feast periods and the dissolved oxygen (DO) time profiles throughout the cycles remained the same for each reaction cycle. Once at steady state, the SBR was sampled several times during a cycle (e.g., 10–11 90-mL samples) to determine dynamics of the concentrations of glucose or acetate, chemical oxygen demand (COD), NH_4_^+^, glycogen, PHB, TAG, total suspended solids (TSS), volatile suspended solids (VSS), and non-volatile suspended solids. The runs on acetate and glucose were replicated twice and three times, respectively.

### Analytical procedures

The samples obtained to monitor changes in the chemical composition of the culture throughout a cycle were processed as follows. Three subsamples of 5 mL were rapidly filtered through a 0.22-μm filter in order to measure TSS (filter dried for 24 h at 105°C) and VSS (dry filter in an oven for 2 h at 550°C). The filtrates were then stored at −20°C until further analyses. Finally, a few drops of formaldehyde were added to the remaining volume of sample to stop biological activities, and subsamples of the biomass were centrifuged for analysis of glycogen (10 mL), PHB (20 mL), and TAG (20 mL). Biomass centrifuged pellets were stored at −80°C until analysis.

The composition of the filtrate was analysed as follows. COD was measured according to the standard method 5220D [22].Colorimetric measurement of NH_4_^+^ was performed using the Berthelot reaction [23]; acetate and glucose concentrations were determined enzymatically using the K-ACETRM kit (Megazyme International Ireland Ltd., Bray, Ireland) and hexokinase enzymatic colorimetric kit (Sigma-Aldrich, St. Louis, MO), respectively, according to the manufacturer’s instructions. All spectrophotometric measurements were performed using a model SpectraMax5 microplate reader (model; Molecular Devices, LLC, USA).

Biomass pellets were analysed for levels of glycogen, PHB, and fatty acids. Glycogen levels were measured according to Maurer et al. (1997) [24]. Briefly, biomass subsamples were resuspended in sterile deionized water and split into 3 technical replicates. The mixtures were hydrolyzed in 0.6 M HCl at 95°C for 2 h, and then neutralized with KOH and phosphate buffer. Glucose concentrations released from glycogen hydrolysis were measured using the hexokinase enzymatic colorimetric kit (Sigma-Aldrich, St. Louis, MO) [24].

PHB levels were determined as described previously [25] [26]. Briefly, the biomass subsamples were resuspended in sterile deionized water and split to perform 3 technical replicates. The mixtures were hydrolyzed in 0.67 M HCl at 65°C for 2 h, then centrifuged, and the pellet was finally extracted with chloroform at 29°C. The chloroform extracts were dried at room temperature overnight in fresh glass vials, and then sulfuric acid was added to the vials and they were further incubated at 95°C for 20 min. After cooling to room temperature, the amount of PHB was determined with the microplate spectrophotometer at 235 nm. DL-3-Hydroxybutiric acid (Sigma-Aldrich, St Louis, MO) was used as standard.

For fatty acid determination, biomass pellets were lyophilised overnight, and 5–8 mg of lyophilized biomass were methanolysed at 100°C for 2.5 h in 600 μL of chloroform and 600 μL of methanol-15% (v/v) H_2_SO_4_. Trinonadecanoin (Nu-Check Prep Inc., Elysian, MN) was used as quantitative internal standard [27]. The organic phase containing fatty acid methyl esters (FAMEs) was analyzed by using an Agilent 6890N GC system equipped with an Agilent HP-88 column (60 m by 0.25 mm, 0.2 μm thick film, 2 mL/min of helium as carrier gas) and a flame ionization detector at 280°C (Agilent, Mississauga, Canada). A 1-μL sample of organic phase was injected with a 50:1 split ratio (inlet temperature: 250°C). The oven program was: 175°C for 15 min, heat to 220°C at 3°C/min, and then 220°C for 5min. The fatty acids were identified and quantified by comparison to standard FAMEs (F.A.M.E. Mix C14-C22, Supelco, Bellefonte, PA, USA). Finally, the reported TAG concentration was calculated from the measured fatty acid concentrations corrected for the amount of fatty acids estimated to be associated with the cell membrane.

### Analysis of labelled amino acids

*R*. *jostii* RHA1 cultures were labelled with ^13^C by incubating 3.65-mL subsamples of the 2-L SBR steady-state cultures in a vessel of a μ-24 bioreactor (Applikon Biotechnology, The Netherlands), and 1.25 mL of fresh MSM with unlabelled carbon source (either glucose or acetate) was added at the beginning of the test. Depending on the labelling condition, 100 μL of 5.36 g/L ^13^C-bicarbonate was added to the vessel at the beginning of either the feast or famine phases. The dissolved oxygen concentration and temperature of the reactors were controlled at 80–100% and 28°C, respectively, while pH was simply monitored throughout the test and remained at 7.0±0.5. Several samples at the end of the feast and famine phases were harvested by addition of 400 μL of concentrated HCl and centrifugation; solids were washed with deionized water and stored at −80°C until amino acid analysis.

Proteinogenic amino acids of the biomass were analyzed using GC-MS technique according to Nanchen et al. (2007) [28]. Briefly, biomass pellets were washed twice with 0.9% NaCl solution, and then hydrolyzed in 6 M HCl for 24 h at 110°C. Hydrolyzates were dried at 70°C overnight, then dissolved in anhydrous pyridine with 10 mg/mL of methoxyamine hydrochloride, and finally derivatized by adding 70 μL of N-tert-butyldimethylsilyl-N-methyltrifluoroacetamide (TBDMS) and incubating at 70°C for 1 h. Deutrated myristic acid (D_27_-myristic acid) was used as internal standard. GC-MS analyses were performed with an Agilent 5975C mass selective detector coupled to a 7890A gas chromatograph (Agilent Technologies, Santa Clara, CA, USA) fitted with a 7693 auto-sampler and a DB-5MS+DG capillary column (30 m plus 10 m Duraguard^®^ diameter 0.25 mm, film thickness 0.25 mm) (Agilent J & W, Santa Clara, CA, USA). The GC temperature program was: 1 min at 60°C, then 10°C/min ramp to 300°C, and finally 320°C for 10 min. The helium carrier flow rate was held constant at 1.5 mL/min (or a flow rate such that the TBDMS derivative of the D_27_-myristic acid has a retention time of 18 min). The injector and interface to the MS were held at 285°C. When operated in full scan mode, the scan range was 50–700 Da. Mass isotopomer distribution data of the labelled amino acids were produced using the *iMS2Flux* software [29].

### Statistical analyses

Elemental mass balances on the measured conversions was performed to check the consistency of the data and reconcile mass balances [30]. The elemental composition matrices are defined in supplementary material Table A in S1 File. As there were more conversions measured than needed to define all elemental balances, a χ^2^ test at 99% confidence level (corresponding to α level of 0.01) was used to evaluate the accuracy of reconciled conversion rates and determine the presence of gross-measurement errors [30].The software Macrobal developed by Hellinga et al. (1992) [30] was used for the calculations.

Using a Bayesian-based objective function discrimination method [31], all the experimentally observed and simulated storage fluxes were combined to evaluate the relative probability of each objective function for the prediction of the mixture of storage compounds in the feast-famine growth (i.e., by comparing the corresponding posterior probability shares).

### Flux balance analysis (FBA)

The recently published *in silico* genome-scale metabolic network of *R*. *jostii* RHA1 (*i*MT1174) was used throughout the study [16]. This model was studied quantitatively by setting up a series of mass balances around each metabolite that can be expressed in matrix notation as: $\frac{dX}{dt}=S.v$; where **x** is the (m×1) vector of the concentrations of the balanced metabolites (i.e., intracellular and macromolecular compounds), **v** denotes the (n×1) vector of the entire metabolic fluxes, and **S** stands for the m×n stoichiometric matrix. The model was then solved by assuming that the cellular network is at steady-state, which simplifies the previous equation to **S.v** = **0**. Because the number of fluxes exceeded the number of metabolites (n > m), the problem is said to be underdetermined. In this case, there is a solution space for the fluxes that can be studied by optimizing proper objective functions subject to defined constraints (the mass balance equations as the physicochemical constraints and the inequalities as the enzymatic capacity of the biochemical reactions) [32].

Objective functions (optimization criteria) need to be defined in order to simulate metabolic behaviour using the underdetermined stoichiometric network. Although various objective functions exist [33], the maximization of the growth rate was reported to be successful at predicting experimental observations for bacteria [15, 34, 35]. It was hypothesized that in typical environmental conditions, biomass formation is typically priorities by the microbial cells; therefore, maximizing the cellular growth rate was defined as the main objective function. In addition, we developed a nested optimization algorithm to consider sub-objective functions. Two sub-objective functions were evaluated: (1) minimization of the sum of absolute values (i.e., the Manhattan norm) of all fluxes [14] (further referred to as minimization of metabolic fluxes), and (2) minimization of metabolic adjustment (further referred to as *environmental* MOMA) defined as minimizing the Manhattan norm of flux differences between feast and famine phases (i.e., |**v**_**Feast**_ **− v**_**Famine**_|_1_) [15].

Simulating the dynamic metabolic behaviour during the feast-famine cycles, a simplified scenario need to be conceptualized. Consequently, each growth phase (i.e., feast or famine) was simulated with a single distribution of average reaction fluxes. According to our experimental observations, we assumed that the feast phase lasted 38% or 40% of the cycle time when glucose or acetate, respectively, were the substrates. Finally, the mass balances on the storage compounds were constrained by equating the total storage synthesis in the feast phase and the consumption in the famine phase.

Using this scenario, we proceeded to map the flux solutions in the space described by the coordinates of substrate uptake rates and storage metabolic fluxes. To simulate the *R*. *jostii* RHA1 growth condition, the substrate uptake rates were fixed to experimentally observed values, and a wide range of storage compound flux combinations simulated. From the simulation results, cycle growth rates were calculated as the time length-weighted averages of the two phases.

### ^13^C-Metabolic flux analysis

To estimate the reactions involved in central metabolism using ^13^C-MFA, a model represents the central metabolism was set-up, which included glycolysis and gluconeogenesis, Entner–Doudoroff pathway, tricarboxylic acid (TCA) cycle, pentose phosphate pathway, anaplerotic carboxylation and decarboxylation, storage metabolic reactions, amino acid biosynthetic reactions, and anabolic routes into biomass (Fig A in S1 File). The openFLUX software application under MATLAB environment (Mathworks Inc, Massachusetts) was used to solve for the fluxes [36]. The following inputs were used for model simulations: cellular composition data of *R*. *jostii* RHA1 defined in *i*MT1174 [16], observed rates of substrate uptake, growth, and storage formation, and mass isotopomer distribution data of the labelled amino acids.

## Results and discussion

### Metabolite pools’ dynamics during feast and famine phases

**Fig 1:**
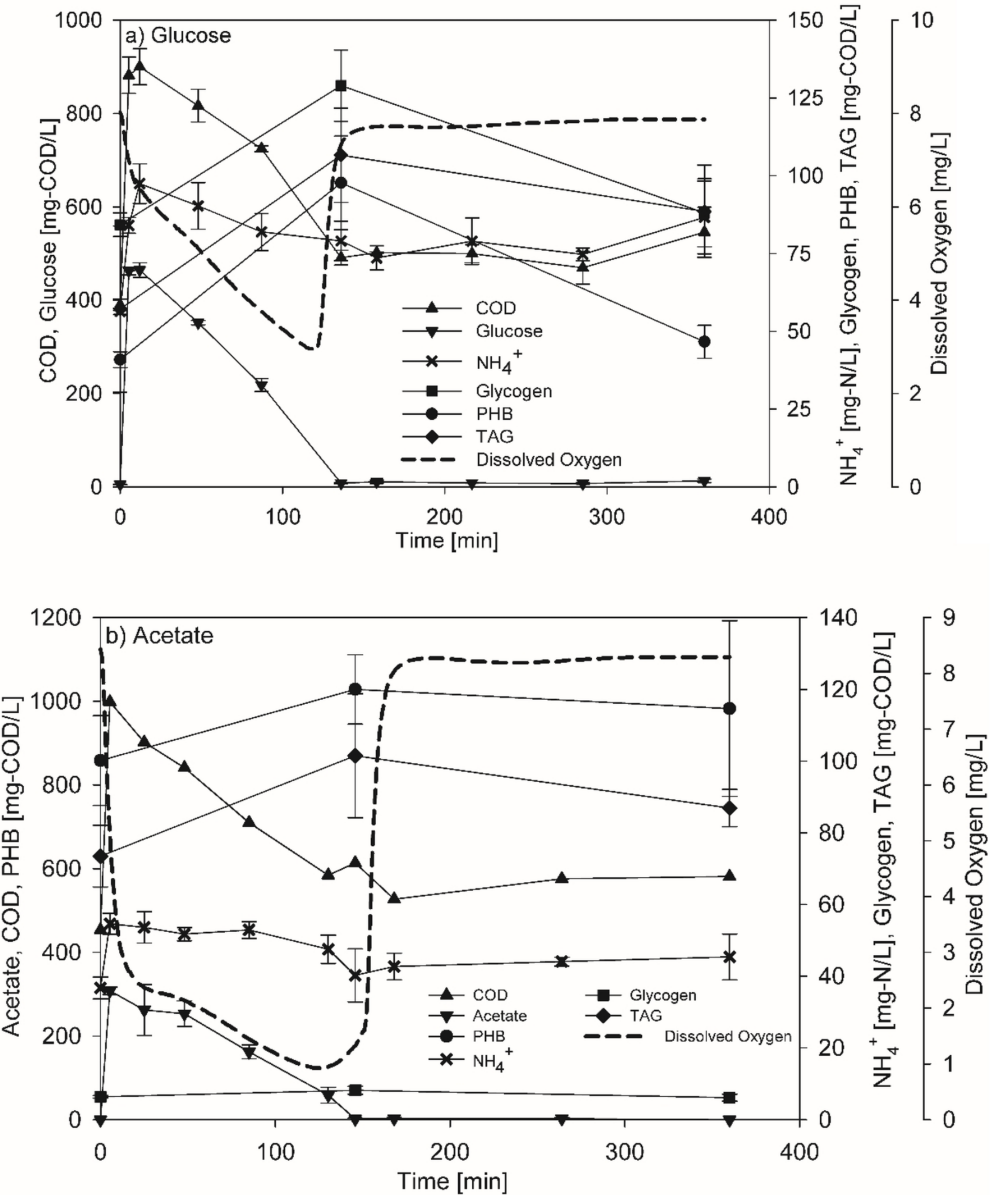
**Dynamics in concentrations of metabolite pools for cultures of *R*. *jostii* RHA1 during 6-hour cycle of SBRs fed glucose (a) or acetate (b) once steady-state was achieved.** SRT was 1 day. Except for DO which are representative profiles, the data points are average of triplicate or duplicate runs for glucose (a) or acetate (b), respectively. Error bars are standard errors. The COD background due to EDTA was measured to be 501.7±12.4 and 576.5±14.1 mg-COD/L for glucose (a) and acetate (b), respectively.

The analysis of the metabolite pools’ dynamics was performed when the reactors were at steady-state, which was defined as a recurrent DO profile in each cycle. The cultures generally reached it five days after inoculation. The DO profiles during the SBR cycles were characterized by a rapid decrease in DO when the carbon substrate was added and a rapid increase when the substrate was completely consumed (Fig 1); thus, this dynamic DO period corresponded to the feast phase. The DO remained relatively high throughout the famine phase. Due to differences in biomass concentrations and specific uptake rates, the feast phase lasted 136.7±6.4 and 145.7±0.0 min on glucose and acetate, respectively, which represented about 38% and 40% of the total cycle.

During the feast phase, linear decreases in glucose or acetate concentrations were observed, indicating a zero-order rate for substrate uptake (Fig 1). At the same time, the concentrations of glycogen, PHB, and TAG increased; however, the accumulation profile was dependent on the carbon substrate. While the three storage compounds were accumulated in similar fashions when glucose was the substrate (Fig 1A), almost no glycogen accumulation was observed on acetate (Fig 1B). As depicted in Fig 1A and 1B, ammonium levels were fairly high at the end of the famine phase indicating unlimited growth conditions on both substrates. This also confirmed that the presence of ammonium during the feast phase did not affect the maximum storage accumulation, as it was observed for activated sludge microbial communities [37].

The fatty acid contents of *R*. *jostii* RHA1 were essentially the same between growth on glucose and acetate (Table B in S1 File). On both substrates, hexadecanoic acid (Palmitic acid, C16:0) was the predominant fatty acid present in TAG followed by cis-octadecenoic acid (C_18:1_); which is consistent with data available for other members of the genus *Rhodococcus* [38, 39].

Given the amount of information obtained on the cultures (substrate [glucose or acetate], biomass, glycogen, PHB, TAG, NH_4_^+^, CO_2_, and DO), it was possible to do elemental mass balances on the measured conversions to check the consistency of the data (Tables C and D in S1 File). In addition to data consistency, the biomass production during the feast and famine phases could be calculated; these data could not be estimated directly from VSS and storage or NH_4_^+^ concentrations because of the small changes in concentrations compared to the background. For both glucose and acetate, it was estimated that approximately 60% of the biomass was synthesized during the feast phase and that 40% was synthesized in the famine phase (Tables C and D in S1 File).

The specific glucose uptake rate on a COD basis was almost 3 times the acetate uptake rate (Table 1), while the overall average biomass yields of *R*. *jostii* RHA1 during the feast-famine cycle on glucose and acetate were 0.25±0.04 and 0.45±0.08 g-COD/g-COD-Substrate, respectively (Table 1). The average rates of glycogen, PHB, and TAG production were similar when glucose was the substrate; however, when acetate was the substrate, the average PHB production rate was 3 times higher than the one of TAG, while glycogen was virtually not accumulated (Table 1).

**Table 1:** Reconciled^a^ specific conversion rates and storage and biomass yields of *R*. *jostii* RHA1 during the feast-famine growth cycle.

Conversion	Units	Glucose	Acetate
**Feast**			
**Substrate uptake rate**	g-COD/(g-COD-Biomass·d)	14.2±2.3^e^	4.9±0.7
**Growth rate**	d^−1^	2.1±0.9	1.4±0.5
**Glycogen synthesis rate**	g-COD/(g-COD-Biomass·d)	1.3±0.3	0.03±0.01
**PHB synthesis rate**^**b**^	g-COD/(g-COD-Biomass·d)	1.6±0.4	0.90±0.57
**TAG synthesis rate**^**c**^	g-COD/(g-COD-Biomass·d)	1.1±0.6	0.32±0.21
**Biomass yield**	g-COD/g-COD-Substrate	0.15±0.06	0.28±0.10
**Glycogen yield**	g-COD/g-COD-Substrate	0.09±0.02	0.01±0.00
**PHB yield**	g-COD/g-COD-Substrate	0.11±0.02	0.18±0.11
**TAG yield**	g-COD/g-COD-Substrate	0.08±0.04	0.07±0.04
**Famine**^**d**^			
**Growth rate**	d^−1^	0.74±0.48	0.52±0.35
**Biomass yield**	g-COD/g-COD-Storage	0.35±0.23	0.68±0.54
**Overall**			
**Overall growth rate**	d^−1^	1.2±0.6	0.87±0.44
**Overall Biomass yield**	g-COD/g-COD-Substrate	0.25±0.04	0.45±0.08

^a^ The reconciled conversion rates and yields were derived using the reconciled converted masses reported in Tables C and D in S1 File.

^b^ Poly-β-hydroxybutyrate.

^c^ Triacylglycerol.

^d^ The storage accumulation rates estimated for the feast phase are the same as their degradation rates in the famine phase.

^e^ ± Reconciled standard error.

These data are in line with previous observations suggesting that the composition of storage compound mixtures accumulated by *R*. *jostii* RHA1 depends on the substrate utilized [18]. However, it is possible that the reactor conditions such as the cycle length and the solids residence time may also play a role; although, it was beyond the scope of the current study to evaluate this possibility. Indeed, work by other groups on activated sludge communities fed glycogen as the sole carbon and energy source found that polyhydroxyalkanoates (PHA) was the main storage compound accumulated at long cycle length (24 h), while glycogen was the main storage at short cycle length (6 h), even though the same microbial populations dominated the communities in both cases [37]. Further studies should address this remaining question.

### FBA to predict storage metabolic fluxes during feast-famine cycles

The capability of FBA to predict storage metabolic fluxes during feast-famine cycles was investigated. Using the experimental data obtained for comparison purposes, the fluxes for the synthesis (feast) and consumption (famine) of glycogen, PHB, and TAG, were simulated using the maximization of the growth rate objective function as the main objective function in conjunction with 2 sub-objective functions: (1) minimizing the Manhattan norm (sum of absolute) of all reaction fluxes (i.e., |**v**|_1_, minimization of fluxes, or minFluxes) [14], (2) minimization of metabolic adjustment between the feast and famine periods (*environmental* MOMA) [15].

For a given substrate uptake rate (here the one experimentally observed), the storage fluxes were highly sensitive to the growth- and non-growth-associated maintenance energy parameters (GAM and NGAM, respectively) and to the sub-objective functions used (Fig B in S1 File). Therefore, the GAMs and NGAMs were adjusted with the aim that the total storage fluxes in g-COD/(g-COD-Biomass·d) corresponded to the experimental ones for a given total growth rate (i.e., SRT). However, it was not possible to fit exactly the total fluxes, and the GAM and NGAM values producing the closest total storage flux simulations were used. The GAM and NGAM values used for each set of simulations are reported in Table 2.

**Table 2:** Comparison of the experimentally observed and simulated storage fluxes (in g-COD/(g-COD-Biomass·d)) at the optimum levels of the two sub-objective functions examined in the feast-famine growth of *R*. *jostii* RHA1 on glucose and acetate as the sole carbon sources.

Substrate	Parameters	Experimental^b^	maxYield^c^	minFluxes^d^	MOMA^e^
		g-COD/(g-COD-Biomass·d)			
**Glucose**	**Substrate**^**a**^	14.2±2.3^f^			
	**Glycogen**	1.3±0.3	0–5.3	1.6^g^	1.3^h^
	**PHB**	1.6±0.4	0–9.7	2.0^g^	1.6^h^
	**TAG**	1.1±0.6	0–8.5	0.6^g^	1.4^h^
**Acetate**	**Substrate**^**a**^	4.9±0.7			
	**Glycogen**	0.03±0.01	0–1.0	0.0^i^	0.0^j^
	**PHB**	0.90±0.57	0–2.9	0.0^i^	1.1^j^
	**TAG**	0.32±0.21	0–2.5	1.5^i^	0.3^j^
**Posterior Probability Share (%)**				1.405×10^−3^	99.999

^a^ Substrate uptake rates are in g-COD/(g-COD-Biomass·d).

^b^ Experimentally observed biosynthetic fluxes of the storage compounds (in g-COD/(g-COD-Biomass·d)).

^c^ The values under the maxYield objective function are its optimal range (minimum value—maximum value in Cmol/Cmol).

^d^ Minimizing the Manhattan norm of the flux vectors while maximizing the growth rate (in mmol/(g-DW·h)).

^e^ Minimizing the Manhattan norm of the difference between the fluxes over the feast-famine cycle while maximizing their growth rates (in mmol/(g-DW·h)) (note that minimizing both the Manhattan norm of the flux vectors and their difference over the feast-famine cycle while maximizing their growth rates showed identical results as compared to *environmental* MOMA).

^f^ ± Reconciled standard error.

^g^ For the minimization of metabolic fluxes sub-objective function on glucose, GAM and NGMA values were set to 410.0 mmol-ATP/g-DW and 10.0 mmol/(g-DW·h), respectively.

^h^ For the *environmental* MOMA sub-objective function on glucose, GAM and NGMA values were set to 475.0 mmol-ATP/g-DW and 7.0 mmol/(g-DW·h), respectively.

^i^ For the minimization of metabolic fluxes sub-objective function on acetate, GAM and NGMA values were set to 0.1 mmol-ATP/g-DW and 1.0 mmol/(g-DW·h), respectively.

^j^ For the *environmental* MOMA sub-objective function on acetate, GAM and NGMA values were set to 1.5 mmol-ATP/g-DW and 2.3 mmol/(g-DW·h), respectively.

**Fig 2:**
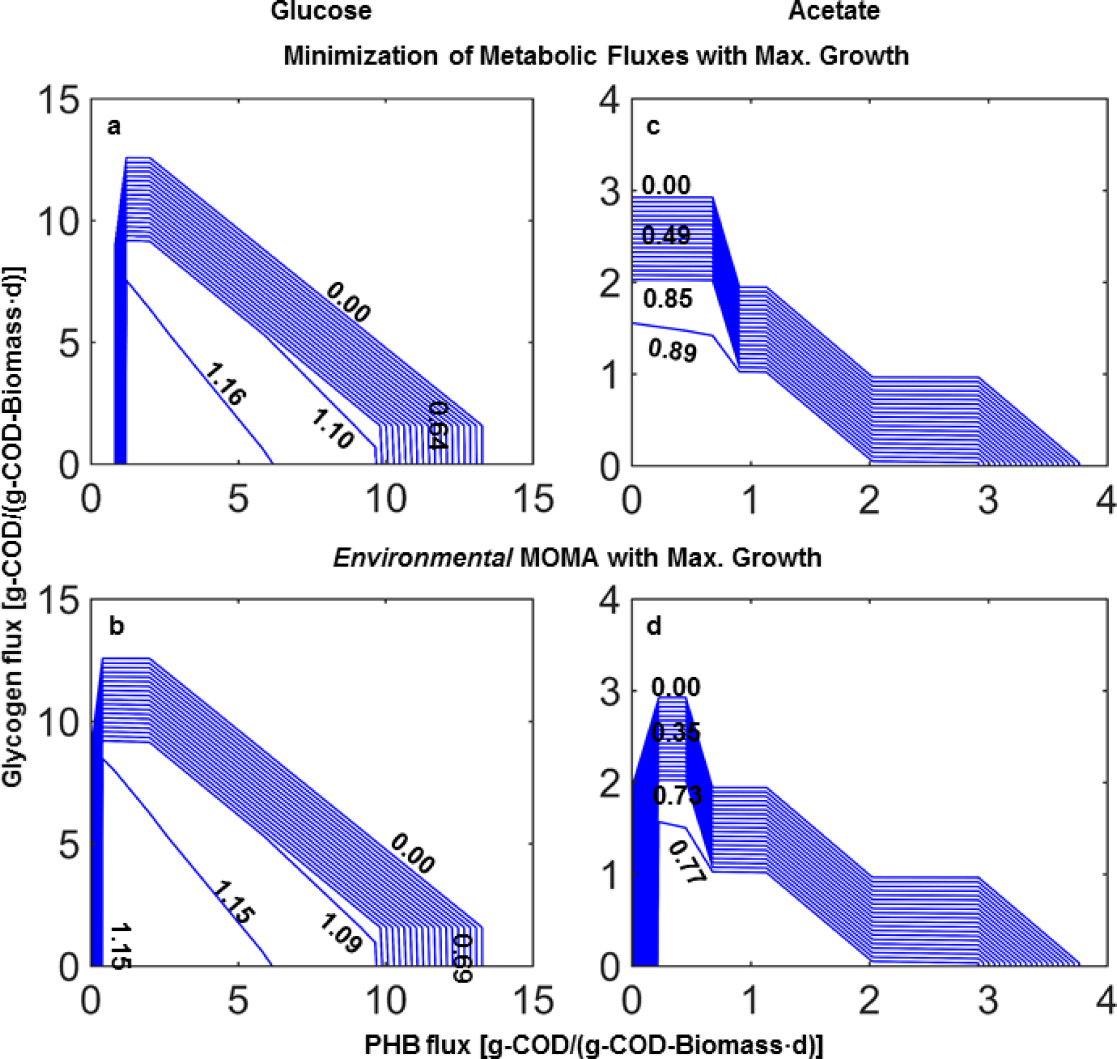
**Contour plots of flux balance results obtained with the two sub-objective functions for weighted average maximum growth rates (isolines, units d-1) in function of pairs of glycogen and PHB storage fluxes at experimentally observed glucose—panels a and b: 14.2 g-COD/(g-COD-Biomass·d)—and acetate uptake rates -panels c and d: 4.9 g-COD/(g-COD-Biomass·d).** TAG fluxes were adjusted to experimentally observed values—panels a and b: 1.1 g-COD/(g-COD-Biomass·d), and panels c and d: 0.3 g-COD/(g-COD-Biomass·d).

The first objective function investigated to assess the metabolic value of the storage compounds was the maximization of the growth rate alone. This is equivalent to yield maximization at low growth rates when organic carbon is not secreted. However, this objective function alone predicted that a range of flux combinations through the storage pools would be optimal, which was not satisfactory for our investigation. Thus, the two sub-objective functions were added. Fig 2 and Figs C and D in S1 File show the contour plots of the total maximum growth rate of *R*. *jostii* RHA1 as a function of set storage fluxes for three different storage fluxes (glycogen, PHB, and TAG) with glucose and acetate as substrate. It can be seen that the total maximum growth rate decreases with increasing storage fluxes (Fig 2), which is due to the metabolic cost of storage transformations.

Based on the flux balance simulations and the optimality of the specific sub-objective functions, the fluxes through each of the storage pools were predicted for the appropriate growth rate (Table 2). The fluxes through all the storage pools were better by the *environmental* MOMA sub-objective function predicted than by the minimization of fluxes sub-objective. For growth on glucose, the trends in fluxes through the storage pools were similarly predicted by the two sub-objective functions, but the absolute difference with the measured date were lower for the *environmental* MOMA. For growth on acetate, minimization of fluxes sub-objective function predicted that only the TAG pool would exhibit significant fluxes, which departed from the observations, while the *environmental* MOMA correctly predicted the flux trends (Table 2). To quantitatively address these relative value of the predictions from simulations with both sub-objective functions, the posterior probability shares of each function were calculated using a Bayesian-based objective function discrimination method [31]. The posterior probability share of *environmental* MOMA sub-objective function was found to be near 1, while one for the minimization of metabolic fluxes was near 0 (Table 2).

The simulation results showed that the *environmental* MOMA sub-objective function predicted the storage fluxes better than the minimization of fluxes sub-objective function on both substrates. Therefore, it would follow that the microorganism optimizes the use of its network such that the reaction fluxes in the famine phase would be similar to the ones in the feast phase. This was further tested by comparing the fluxes of the central metabolic network of RHA1 in the feast and famine phase estimated by the ^13^C-MFA (next section).

### Central metabolism reaction fluxes during feast-famine cycles

**Fig 3:**
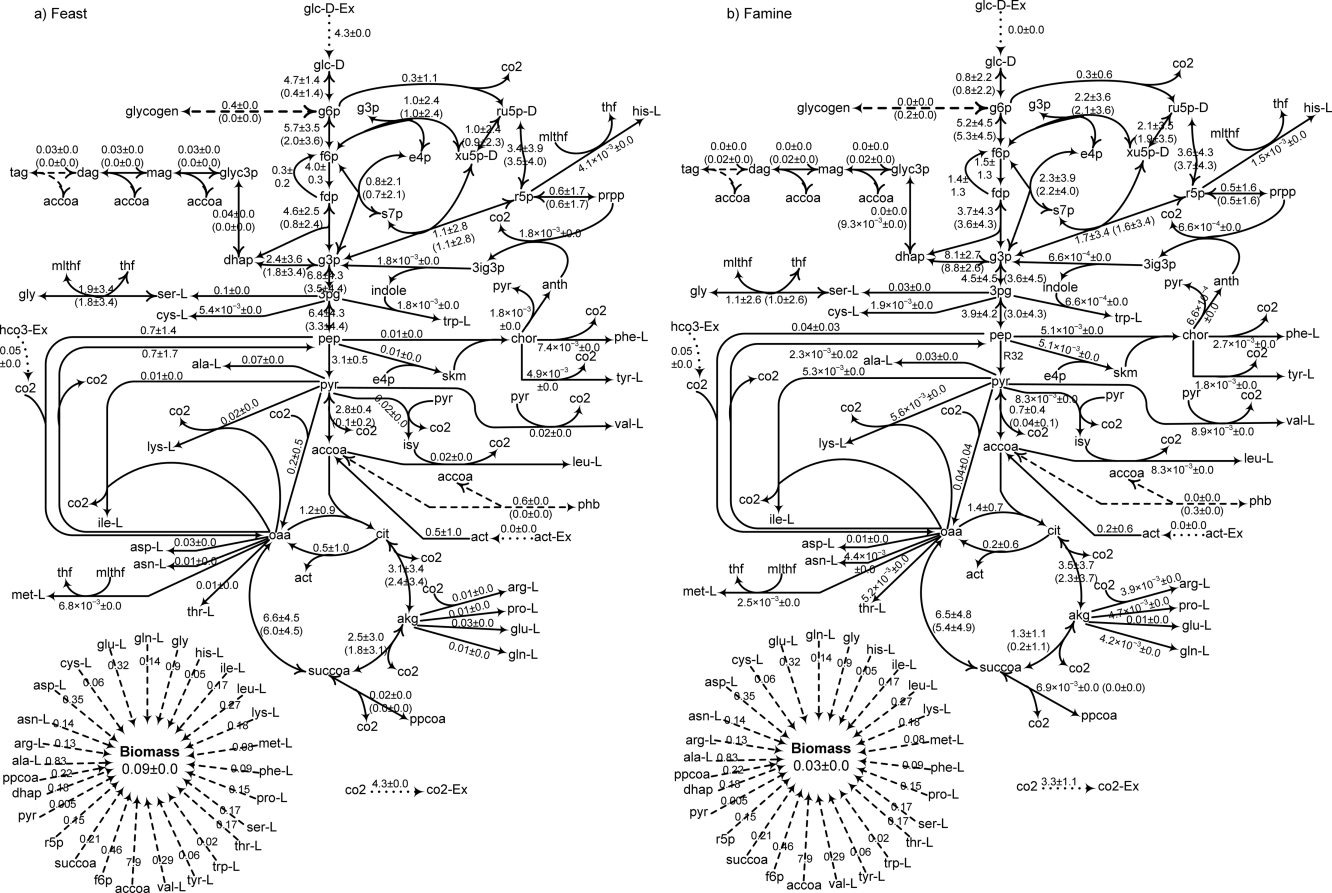
**Fluxes of the central metabolic reactions of *R*. *jostii* RHA1 in the feast phase (left panel) or the famine for growth on glucose.** Unlabelled glucose was added at the beginning of the feast phase, and ^13^C-bicarbonate was added either at the beginning of the feast or the famine phase. Reported fluxes are the average of 100 solutions ± the corresponding standard deviations.

**Fig 4:**
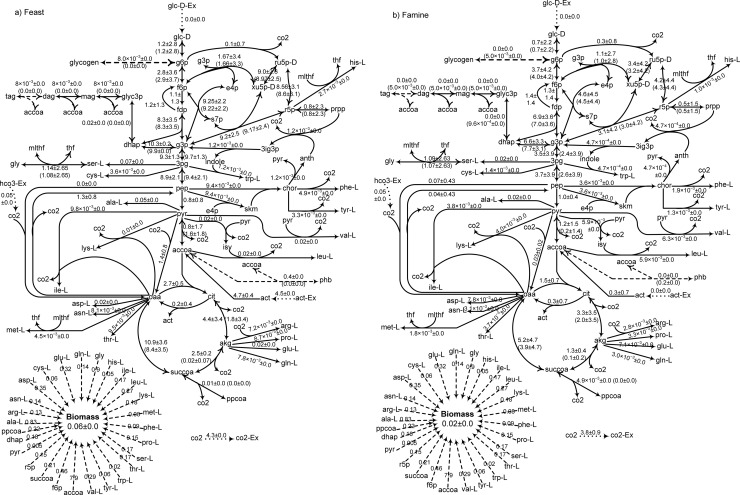
**Fluxes of the central metabolic reactions of *R*. *jostii* RHA1 in the feast phase (left panel) or the famine for growth on acetate.** Unlabelled acetate was added at the beginning of the feast phase, and ^13^C-bicarbonate was added either at the beginning of the feast or the famine phase. Reported fluxes are the average of 100 solutions ± the corresponding standard deviations.

The fluxes through the central metabolism reactions were characterized using ^13^C-MFA with addition of ^13^C-bicarbonate as the labelled carbon source. *R*. *jostii* RHA1 was fed with unlabelled glucose or acetate, and ^13^C-bicarobnate was added to the medium at the beginning of either the feast or famine phase. The ^13^C-MFA solution of the models was obtained by adjusting the metabolic fluxes that were experimentally determined to their measured values. Additionally, the effective ^13^C-bicarbonate uptake rate was unknown. Thus, ^13^C-bicarbonate uptake rate was adjusted by minimizing the sum of absolute differences between the calculated and experimentally observed mass isotopomer distribution (i.e., the error) vectors in all cases. The optimal value of ^13^C-bicarbonate uptake rate was found to be 0.05 mmol/(g-DW·h) by this method. The average fluxes determined from 100 simulations yielding the closest fit to the observed mass isotopomer distributions are presented in Figs 3 and 4. They show the flux distributions through the central metabolism were similar in the feast and the famine phases.

**Fig 5:**
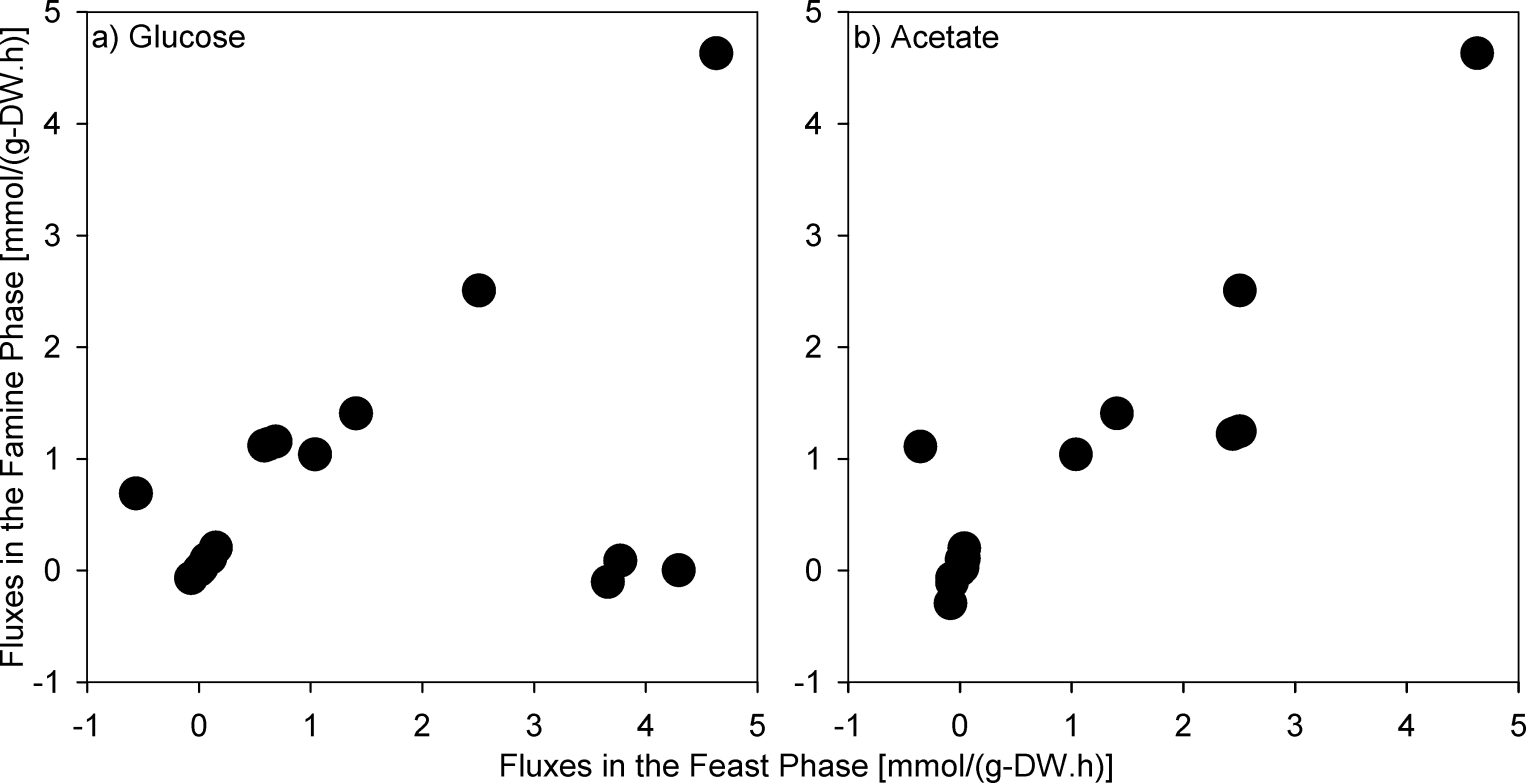
Correlation plots of the 19 central metabolism reactions of *R*. *jostii* RHA1 in the feast and famine phases estimated using ^13^C-MFA on glucose (a) and acetate (b).

To help with the visualization, the estimated fluxes of the 19 central metabolism reactions in the feast were plotted vs. the one obtained for the famine phase (Fig 5). On glucose (Fig 5A), it can be seen that some reactions are virtually not used during the famine phase (high fluxes in the feast phase, but near zero during the famine phase). These reaction corresponds to R5(R6) (KEGG ID:R01786, ATP:α-D-glucose 6-phosphotransferase), R7(R8) (KEGG ID: R02740, α-D-Glucose 6-phosphate ketol-isomerase), and R11(R12) (KEGG ID: R01070, β-D-fructose-1,6-bisphosphate D-glyceraldehyde-3-phosphate-lyase). To the exception of these reactions, the fluxes through central metabolic reactions during the famine phase a generally linearly related to the ones for the same reactions observed during the feast phase when both glucose and acetate are the substrate. This result supports the hypothesis behind the *environmental* MOMA sub-objective function stating that the organism minimizes the difference in fluxes between the feast and famine phases.

## Conclusions

In this study, the storage metabolism of *R*. *jostii* RHA1 was investigated during steady-state feast-famine cycles on glucose and acetate as the sole carbon sources by means of its genome-scale metabolic model and the FBA approach, and it was experimentally validated. *R*. *jostii* RHA1 is able to simultaneously accumulate three storage compounds (PHB, TAG, and glycogen). It was experimentally observed that when glucose was the substrate, the three storage compounds exhibited similar COD accumulation; however, when acetate was the substrate, PHB accumulation turned out to be 3 times greater than that of TAG, while glycogen was essentially not accumulated. The genome-scale metabolic model of *R*. *jostii* RHA1 (*i*MT1174) and FBA were employed in order to define a suitable objective function capable of predicting these phenotypic behaviours. To this end, maximization of the growth rate was set as the main objective function, while minimization of total reaction fluxes and *environmental* MOMA were implemented as the sub-objective functions because the maximization of the growth rate alone did not precisely constrained the storage fluxes. The *environmental* MOMA sub-objective function better predicted the mixture of storage compounds accumulated as compared to the minimization of total reaction fluxes sub-objective function. In addition, analyses of ^13^C-labelling experiments with ^13^C-MFA found results in line with the fundamental *environmental* MOMA hypothesis that the fluxes through the central metabolic network of *R*. *jostii* RHA1 during the feast phases were similar to the ones during the famine phases. Together, these results support the *environmental* MOMA sub-objective function could predict cellular metabolism in cyclical environmental conditions such as the feast and famine conditions.

## Supporting information

S1 File Supporting information. Including: **Table A**. Elemental composition matrix for flux data reconciliation. **Table B**. Fatty acid content of *R*. *jostii* RHA1 at the beginning and end of the feast phase on glucose and acetate. **Table C**. Comparison between measured and reconciled converted masses on glucose in a steady-state feast-famine cycle. **Table D**. Comparison between measured and reconciled converted masses on acetate in a steady-state feast-famine. **Fig A** The central metabolic pathways of *R*. *jostii* RHA1, along with the amino acid, storage, and biomass biosynthetic reactions. **Fig B.** Contours of the total storage flux [g-COD/(g-COD-Biomass·d)] as a function of the growth- and non-growth- associated maintenance energy (GAM and NGAM, respectively) for the simulated feast growth with glucose (a, b, and c) or acetate (d, e, and f) as carbon substrates. **Fig C.** Contour plots of flux balance results obtained with the minimization of metabolic fluxes sub-objective function in conjunction with the maximization of growth rate for weighted average maximum growth rates (isolines, units d^-1^) in function of pairs of glycogen and PHB (a and d), PHB and TAG (b and e), and TAG and glycogen (c and f) storage fluxes at experimentally observed glucose—panels a, b, and c: 14.2 g-COD/(g-COD-Biomass·d)—and acetate uptake rates—panels d, e, and f: 4.9 g-COD/(g-COD-Biomass·d). **Fig D.** Contour plots of flux balance results obtained with the *environmental* MOMA sub-objective function in conjunction with the maximization of growth rate for weighted average maximum growth rates (isolines, units d^-1^) in function of pairs of glycogen and PHB (a and d), PHB and TAG (b and e), and TAG and glycogen (c and f) storage fluxes at experimentally observed glucose—panels a, b, and c: 14.2 g-COD/(g-COD-Biomass·d)—and acetate uptake rates—panels d, e, and f: 4.9 g-COD/(g-COD-Biomass·d).
(DOCX)
