# Supplementary material for: Predicting the accumulation of storage compounds by Rhodococcus jostii RHA1 in the feast-famine growth cycles using genome-scale flux balance analysis
Source: PLoS One. 2018 Mar 1;13(3):e0191835. doi: 10.1371/journal.pone.0191835 (PMC5832212; doi:10.1371/journal.pone.0191835)
Supplement: S1 File — Including: Table A. Elemental composition matrix for flux data reconciliation. Table B. Fatty acid content of R. jostii RHA1 at the beginning and end of the feast phase on glucose and acetate. Table C. Comparison between measured and reconciled converted masses on glucose in a steady-state feast-famine cycle. Table D. Comparison between measured and reconciled converted masses on acetate in a steady-state feast-famine. Fig A The central metabolic pathways of R. jostii RHA1, along with the amino acid, storage, and biomass biosynthetic reactions. Fig B. Contours of the total storage flux [g-COD/(g-COD-Biomass·d)] as a function of the growth- and non-growth- associated maintenance energy (GAM and NGAM, respectively) for the simulated feast growth with glucose (a, b, and c) or acetate (d, e, and f) as carbon substrates. Fig C. Contour plots of flux balance results obtained with the minimization of metabolic fluxes sub-objective function in conjunction with the maximization of growth rate for weighted average maximum growth rates (isolines, units d-1) in function of pairs of glycogen and PHB (a and d), PHB and TAG (b and e), and TAG and glycogen (c and f) storage fluxes at experimentally observed glucose—panels a, b, and c: 14.2 g-COD/(g-COD-Biomass·d)—and acetate uptake rates—panels d, e, and f: 4.9 g-COD/(g-COD-Biomass·d). Fig D. Contour plots of flux balance results obtained with the environmental MOMA sub-objective function in conjunction with the maximization of growth rate for weighted average maximum growth rates (isolines, units d-1) in function of pairs of glycogen and PHB (a and d), PHB and TAG (b and e), and TAG and glycogen (c and f) storage fluxes at experimentally observed glucose—panels a, b, and c: 14.2 g-COD/(g-COD-Biomass·d)—and acetate uptake rates—panels d, e, and f: 4.9 g-COD/(g-COD-Biomass·d). (DOCX) [file pone.0191835.s001.docx]

# Supporting Information

# Predicting the Accumulation of Storage Compounds by *Rhodococcus jostii* RHA1 in the Feast-famine Growth Cycles Using Genome-scale Flux Balance Analysis

Mohammad Tajparast^1^ and Dominic Frigon^1*^

^1^Microbial Community Engineering Laboratory, Department of Civil Engineering and Applied Mechanics, McGill University, 817 Sherbrooke Street West, Montreal, Quebec, Canada, H3A 0C3

^*^Corresponding author

Email addresses:

MT: [mohammad.tajparast@mail.mcgill.ca](mailto:mohammad.tajparast@mail.mcgill.ca)

DF: dominic.frigon@mcgill.ca

**Short Title:** Genome-scale Flux Balance of RHA1 during Feast-famine Cycles

**Table A.** Elemental composition matrix for flux data reconciliation.

| **Conversion** | **Feast Balance** | | | **Famine Balance** | | | **Feast-Famine Equality** | | | **Overall**  **Biomass** |
| --- | --- | --- | --- | --- | --- | --- | --- | --- | --- | --- |
|  | **C^d^** | **N^e^** | **COD^f^** | **C** | **N** | **COD** | **Glycogen** | **PHB** | **TAG** |  |
| **Feast** |  |  |  |  |  |  |  |  |  |  |
| **Substrate** | 0.375 | 0 | 1 | 0 | 0 | 0 | 0 | 0 | 0 | 0 |
| **NH_4_^+^** | 0 | 1 | 0 | 0 | 0 | 0 | 0 | 0 | 0 | 0 |
| **Glycogen** | 0.375 | 0 | 1 | 0 | 0 | 0 | 1 | 0 | 0 | 0 |
| **PHB^a^** | 0.333 | 0 | 1 | 0 | 0 | 0 | 0 | 1 | 0 | 0 |
| **TAG^b^** | 0.266 | 0 | 1 | 0 | 0 | 0 | 0 | 0 | 1 | 0 |
| **Biomass** | 0.320 | 0.073 | 1 | 0 | 0 | 0 | 0 | 0 | 0 | 1 |
| **O_2_** | 0 | 0 | −1 | 0 | 0 | 0 | 0 | 0 | 0 | 0 |
| **CO_2_** | 1 | 0 | 0 | 0 | 0 | 0 | 0 | 0 | 0 | 0 |
| **Famine** |  |  |  |  |  |  |  |  |  |  |
| **NH_4_^+^** | 0 | 0 | 0 | 0 | 1 | 0 | 0 | 0 | 0 | 0 |
| **Glycogen** | 0 | 0 | 0 | 0.375 | 0 | 1 | 1 | 0 | 0 | 0 |
| **PHB** | 0 | 0 | 0 | 0.333 | 0 | 1 | 0 | 1 | 0 | 0 |
| **TAG** | 0 | 0 | 0 | 0.266 | 0 | 1 | 0 | 0 | 1 | 0 |
| **Biomass** | 0 | 0 | 0 | 0.320 | 0.073 | 1 | 0 | 0 | 0 | 1 |
| **O_2_** | 0 | 0 | 0 | 0 | 0 | −1 | 0 | 0 | 0 | 0 |
| **CO_2_** | 0 | 0 | 0 | 1 | 0 | 0 | 0 | 0 | 0 | 0 |
| **OverallBiomass^c^** | 0 | 0 | 0 | 0 | 0 | 0 | 0 | 0 | 0 | −1 |
| ^a^: Poly-β-hydroxybutyrate; ^b^: triacylglycerol; ^c^: total amount of active biomass produced in one feast-famine cycle; ^d^: carbon; ^e^: nitrogen; ^f^: chemical oxygen demand. | | | | | | | | | | |

**Table B.** Fatty acid content of *R. jostii* RHA1 at the beginning and end of the feast phase on glucose and acetate.

| **Fatty Acid** | **Units** | **Glucose** | |  | **Acetate** | |
| --- | --- | --- | --- | --- | --- | --- |
|  |  | **Beginning Feast** | **End Feast** |  | **Beginning Feast** | **End Feast** |
| **C14:0** | %g-COD/g-COD-TAG | 2.1±1.1^a^ | 3.0±0.2 |  | 3.0±0.2 | 3.0±0.1 |
| **C15:0** | %g-COD/g-COD-TAG | 3.9±1.9 | 5.7±0.2 |  | 6.6±1.8 | 5.4±0.3 |
| **C16:0** | %g-COD/g-COD-TAG | 66.7±16.7 | 48.4±2.4 |  | 47.8±2.3 | 48.5±1.3 |
| **C17:0** | %g-COD/g-COD-TAG | 5.2±2.6 | 7.8±0.1 |  | 8.9±1.5 | 8.0±0.5 |
| **C18:0** | %g-COD/g-COD-TAG | 7.5±4.7 | 13.2±3.4 |  | 6.6±0.0 | 6.8±0.2 |
| **C18:1 Trans** | %g-COD/g-COD-TAG | 0.0±0.0 | 0.0±0.0 |  | 0.0±0.0 | 0.8±0.8 |
| **C18:1 Cis** | %g-COD/g-COD-TAG | 14.6±7.5 | 21.5±0.9 |  | 27.1±1.1 | 27.6±0.1 |
| **C18:2 Trans** | %g-COD/g-COD-TAG | 0.0±0.0 | 0.3±0.3 |  | 0.0±0.0 | 0.0±0.0 |
| **Total TAGs** | %g-COD/g-COD-Biomass | 2.6±1.3 | 4.2±0.6 |  | 4.1±0.1 | 5.9±0.2 |
| ^a^: Average ± standard errors of 3 replicate runs. | | | | | | |

**Table C.** Comparison between measured and reconciled converted masses on glucose in a steady-state feast-famine cycle.

| **Conversion** | **Units** | **Calculation^d^** | **Measurements** | | | |  | **Balance Estimates^g^** | | | | | |
| --- | --- | --- | --- | --- | --- | --- | --- | --- | --- | --- | --- | --- | --- |
|  |  |  | **Conversions** | | **Error** | |  | | **Conversions** | | **Error** | | |
| **Feast** |  |  |  |  | |  | | | |  | | |  |
| **Phase Time** | min |  | 136.7 | 6.4 | | NA | | | | NA | | |  |
| **Biomass Concentration** | mg-COD/L |  | 358.3 | 54.0 | | NA | | | | NA | | |  |
| **ASH Concentration** | mg/L |  | 35.8 | 5.4 | | NA | | | | NA | | |  |
| **Substrate** | mg-COD/cycle | B | −956.3 | 20.8 | | −963.4 | | | | 19.9 | | |  |
| **NH_4_^+^** | mg-N/cycle | B | −37.8 | 13.5 | | −10.3 | | | | 3.9 | | |  |
| **Glycogen** | mg-COD/cycle | B | 89.4 | 24.1 | | 86.0 | | | | 17.0 | | |  |
| **PHB^a^** | mg-COD/cycle | B | 113.7 | 30.6 | | 108.2 | | | | 21.5 | | |  |
| **TAG^b^** | mg-COD/cycle | B | 99.0 | 61.4 | | 76.8 | | | | 42.0 | | |  |
| **Biomass Production** | mg-COD/cycle | C | NA^e^ | NA | | 140.9 | | | | 53.7 | | |  |
| **O_2_** | mg-O_2_/cycle | B | −554.9 | 14.4 | | −551.5 | | | | 14.1 | | |  |
| **CO_2_** | mg-C/cycle | C | NA | NA | | 227.5 | | | | 5.3 | | |  |
| **Famine** |  |  |  |  | |  | | | |  | | |  |
| **Phase Time** | min |  | 223.3 | 6.4 | | NA | | | | NA | | |  |
| **Biomass Concentration** | mg-COD/L |  | 417.5 | 50.2 | | NA | | | | NA | | |  |
| **ASH Concentration** | mg/L |  | 41.8 | 5.0 | | NA | | | | NA | | |  |
| **NH_4_^+^** | mg-N/cycle | B | 0.0^f^ | 27.0^f^ | | −7.0 | | | | 4.3 | | |  |
| **Glycogen** | mg-COD/cycle | B | −89.4 | 24.1 | | −86.0 | | | | 17.0 | | |  |
| **PHB** | mg-COD/cycle | B | −113.7 | 30.6 | | −108.2 | | | | 21.5 | | |  |
| **TAG** | mg-COD/cycle | B | −99.0 | 61.4 | | −76.8 | | | | 42.0 | | |  |
| **Biomass Production** | mg-COD/cycle | C | NA | NA | | 95.8 | | | | 58.6 | | |  |
| **O_2_** | mg-O_2_/cycle | B | −182.2 | 39.1 | | −175.2 | | | | 33.0 | | |  |
| **CO_2_** | mg-C/cycle | C | NA | NA | | 58.1 | | | | 10.8 | | |  |
| **Overall** |  |  |  |  | |  | | | |  | | |  |
| **Overall Biomass Production^c^** | mg-COD/cycle | B | 253.9 | 56.9 | | 236.7 | | | | 35.9 | | |  |
| ^a^: Poly-β-hydroxybutyrate; ^b^: triacylglycerol; ^c^: total amount of active biomass produced in one feast-famine cycle; ^d^: this column indicates whether the compound will be balanced (B), which means that a better estimate will be found, and be calculated (C) using the elemental composition matrix in Table S1; ^e^: not applicable; ^f^: the ammonium uptake rate in the famine phase was set to zero, and its respective error was set twice as much as that of the feast phase; ^g^: note that h = 4.87 against χ^2^ = 16.80 at 99% confidence level that means there is no difference between the measured and reconciled data at 99% confidence. | | | | | | | | | | | |  |  |

**Table D.** Comparison between measured and reconciled converted masses on acetate in a steady-state feast-famine cycle.

| **Conversion** | **Units** | **Calculation^d^** | **Measurements** | | | |  | **Balance Estimates^f^** | | | | | |
| --- | --- | --- | --- | --- | --- | --- | --- | --- | --- | --- | --- | --- | --- |
|  |  |  | **Conversions** | | **Error** | |  | | **Conversions** | | **Error** | | |
| **Feast** |  |  |  |  | |  | | | |  | | |  |
| **Phase Time** | min |  | 145.7 | 0.0 | | NA | | | | NA | | |  |
| **Biomass Concentration** | mg-COD/L |  | 653.8 | 95.0 | | NA | | | | NA | | |  |
| **ASH Concentration** | mg/L |  | 65.4 | 9.5 | | NA | | | | NA | | |  |
| **Substrate** | mg-COD/cycle | B | −643.8 | 25.6 | | −651.5 | | | | 24.8 | | |  |
| **NH_4_^+^** | mg-N/cycle | B | −21.1 | 5.7 | | −13.3 | | | | 4.8 | | |  |
| **Glycogen** | mg-COD/cycle | B | 3.7 | 2.4 | | 3.6 | | | | 1.7 | | |  |
| **PHB^a^** | mg-COD/cycle | B | 340.1 | 157.0 | | 119.7 | | | | 73.0 | | |  |
| **TAG^b^** | mg-COD/cycle | B | 56.0 | 38.6 | | 42.7 | | | | 26.8 | | |  |
| **Biomass Production** | mg-COD/cycle | C | NA^e^ | NA | | 181.1 | | | | 65.7 | | |  |
| **O_2_** | mg-O_2_/cycle | B | −385.5 | 82.8 | | −304.4 | | | | 50.5 | | |  |
| **CO_2_** | mg-C/cycle | C | NA | NA | | 133.8 | | | | 17.0 | | |  |
| **Famine** |  |  |  |  | |  | | | |  | | |  |
| **Phase Time** | min |  | 214.3 | 0.0 | | NA | | | | NA | | |  |
| **Biomass Concentration** | mg-COD/L |  | 727.5 | 91.8 | | NA | | | | NA | | |  |
| **ASH Concentration** | mg/L |  | 72.7 | 9.2 | | NA | | | | NA | | |  |
| **NH_4_^+^** | mg-N/cycle | C | NA | NA | | −8.3 | | | | 5.3 | | |  |
| **Glycogen** | mg-COD/cycle | B | −3.7 | 2.4 | | −3.6 | | | | 1.7 | | |  |
| **PHB** | mg-COD/cycle | B | −340.1 | 157.0 | | −119.7 | | | | 73.0 | | |  |
| **TAG** | mg-COD/cycle | B | −56.0 | 38.6 | | −42.7 | | | | 26.8 | | |  |
| **Biomass Production** | mg-COD/cycle | C | NA | NA | | 113.6 | | | | 72.3 | | |  |
| **O_2_** | mg-O_2_/cycle | B | −51. 6 | 11.7 | | −52.4 | | | | 11.6 | | |  |
| **CO_2_** | mg-C/cycle | C | NA | NA | | 16.3 | | | | 4.2 | | |  |
| **Overall** |  |  |  |  | |  | | | |  | | |  |
| **Overall Biomass Production^c^** | mg-COD/cycle | B | 270.3 | 63.4 | | 294.7 | | | | 48.2 | | |  |
| ^a^: Poly-β-hydroxybutyrate; ^b^: triacylglycerol; ^c^: total amount of active biomass produced in one feast-famine cycle; ^d^: this column indicates whether the compound will be balanced (B), which means that a better estimate will be found, and be calculated (C) using the elemental composition matrix in Table S1; ^e^: not applicable; ^f^: note that h = 7.29 against χ^2^ = 15.1 at 99% confidence level that means there is no difference between the measured and reconciled data at 99% confidence. | | | | | | | | | | | |  |  |


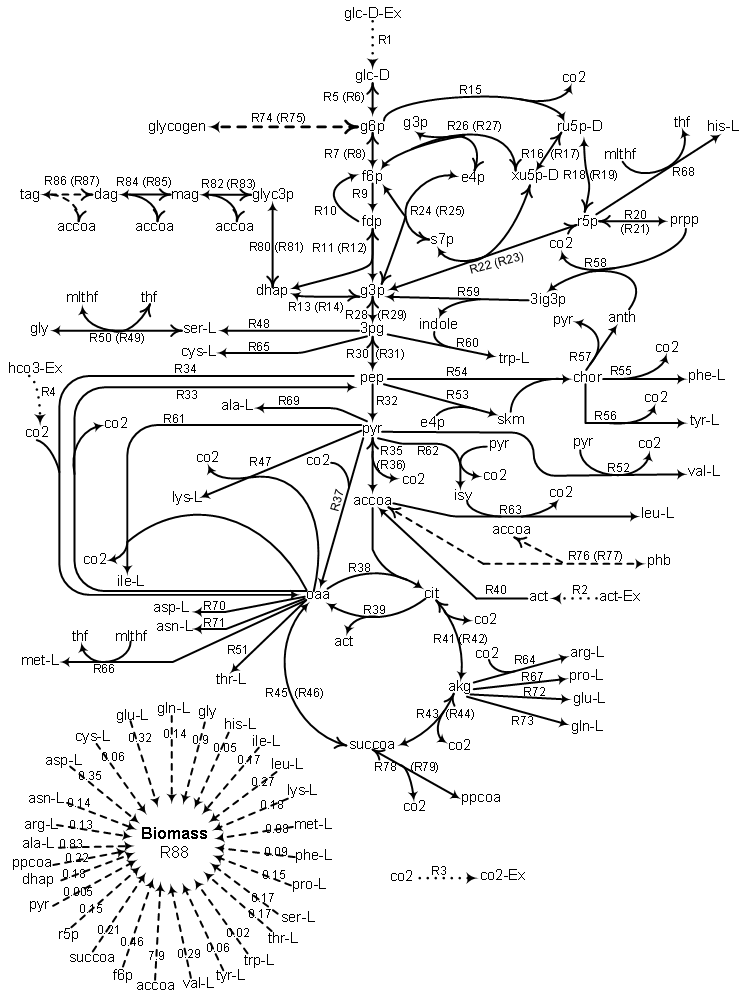


**Fig. A.** The central metabolic pathways of *R. jostii* RHA1, along with the amino acid, storage, and biomass biosynthetic reactions.

Following is the list of the metabolites involved in the above *R. jostii* RHA1 network:

glc-D-Ex: extracellular alpha-D-Glucose, glc-D: alpha-D-Glucose, act-Ex: extracellular acetate, act: acetate, co2-Ex: extracellular carbon dioxide, co2: carbon dioxide, hco3-Ex: extracellular bicarbonate, g6p: alpha-D-Glucose 6-phosphate, f6p: beta-D-Fructose 6-phosphate, fdp: beta-D-Fructose 1,6-bisphosphate, g3p: D-Glyceraldehyde 3-phosphate, dhap: Glycerone phosphate, ru5p-D: D-Ribulose 5-phosphate, xu5p-D: D-Xylulose 5-phosphate, r5p: D-Ribose 5-phosphate, prpp: 5-Phospho-alpha-D-ribose 1-diphosphate, s7p: Sedoheptulose 7-phosphate, e4p: D-Erythrose 4-phosphate, 3pg: 3-Phospho-D-glycerate, pep: Phosphoenolpyruvate, pyr: Pyruvate, oaa: Oxaloacetate, accoa: Acetyl-CoA, cit: Citrate, akg: 2-Oxoglutarate, succoa: Succinyl-CoA, lys-L: L-Lysine, ser-L: L-Serine, gly: Glycine, mlthf: 5,10-Methylenetetrahydrofolate, thf: Tetrahydrofolate, thr-L: L-Threonine, val-L: L-Valine, skm: Shikimate, chor: Chorismate, phe-L: L-Phenylalanine, tyr-L: L-Tyrosine, anth: Anthranilate, 3ig3p: Indoleglycerol phosphate, indole: Indole, trp-L: L-Tryptophan, ile-L: L-Isoleucine, isv: ISV, leu-L: L-Leucine, arg-L: L-Arginine, cys-L: L-Cysteine, met-L: L-Methionine, pro-L: L-Proline, his-L: L-Histidine, ala-L: L-Alanine, asp-L: L-Aspartate, asn-L: L-Asparagine, glu-L: L-Glutamate, gln-L: L-Glutamine, glycogen: Glycogen, phb: Poly-beta-hydroxybutyrate, ppcoa: Propanoyl-CoA, glyc3p: sn-Glycerol 3-phosphate, mag: sn-Glycerol1, dag: sn-Glycerol2, and tag: Triacylglycerol.

Following is the list of the reactions, along with their KEGG IDs, involved in the above *R. jostii* RHA1 network; b in the KEGG IDs stands for the backward reaction.

R1: A00576, R2: A00680, R3: A00251, R4: A00677, R5: R01786, R6: R01786b, R7: R02740, R8: R02740b, R9: R04779, R10: R04780, R11: R01070, R12: R01070b, R13: R01015b, R14: R01015, R15: lumped reaction, R16: R01529, R17: R01529b, R18: R01056b, R19: R01056, R20: R01049, R21: R01049b, R22: R01641, R23: R01641b, R24: R01827, R25: R01827b, R26: R01830, R27: R01830b, R28: R01015, R29: R01015b, R30: lumped reaction, R31: lumped reaction, R32: R00200, R33: R00431, R34: R00345, R35: R01196, R36: R01196b, R37: R00344, R38: R00351, R39: R00362, R40: R00235, R41: lumped reaction, R42: lumped reaction, R43: A00619, R44: A00619b, R45: lumped reaction, R46: lumped reaction, R47: L-Lysine biosynthesis (lumped reaction), R48: L-Serine biosynthesis (lumped reaction), R49: R00945, R50: R00945b, R51: L-Threonine biosynthesis (lumped reaction), R52: L-Valine biosynthesis (lumped reaction), R53: Shikimate biosynthesis (lumped reaction), R54: Chorismate biosynthesis (lumped reaction), R55: L-Phenylalanine biosynthesis (lumped reaction), R56: L-Tyrosine biosynthesis (lumped reaction), R57: R00985, R58: lumped reaction, R59: R02340, R60: R00674, R61: L-Isoleucine biosynthesis (lumped reaction), R62: L-Leucine biosynthesis (lumped reaction), R63: L-Leucine biosynthesis (lumped reaction), R64: L-Arginine biosynthesis (lumped reaction), R65: L-Cysteine biosynthesis (lumped reaction), R66: L-Methionine biosynthesis (lumped reaction), R67: L-Proline biosynthesis (lumped reaction), R68: L-Histidine biosynthesis (lumped reaction), R69: L-Alanine biosynthesis (lumped reaction), R70: L-Aspartate biosynthesis (lumped reaction), R71: L-Asparagine biosynthesis (lumped reaction), R72: L-Glutamate biosynthesis (lumped reaction), R73: L-Glutamine biosynthesis (lumped reaction), R74: R00959b, R75: R00959, R76: R00238, R77: R00238b, R78: R00833, R79: R00833b, R80: R01011, R81: R01011b, R82: Triacylglycerol biosynthesis (lumped reaction), R83: Triacylglycerol biosynthesis (lumped reaction), R84: Triacylglycerol biosynthesis (lumped reaction), R85: Triacylglycerol biosynthesis (lumped reaction), R86: Triacylglycerol biosynthesis (lumped reaction), R87: Triacylglycerol biosynthesis (lumped reaction), and R88: A00000.

**
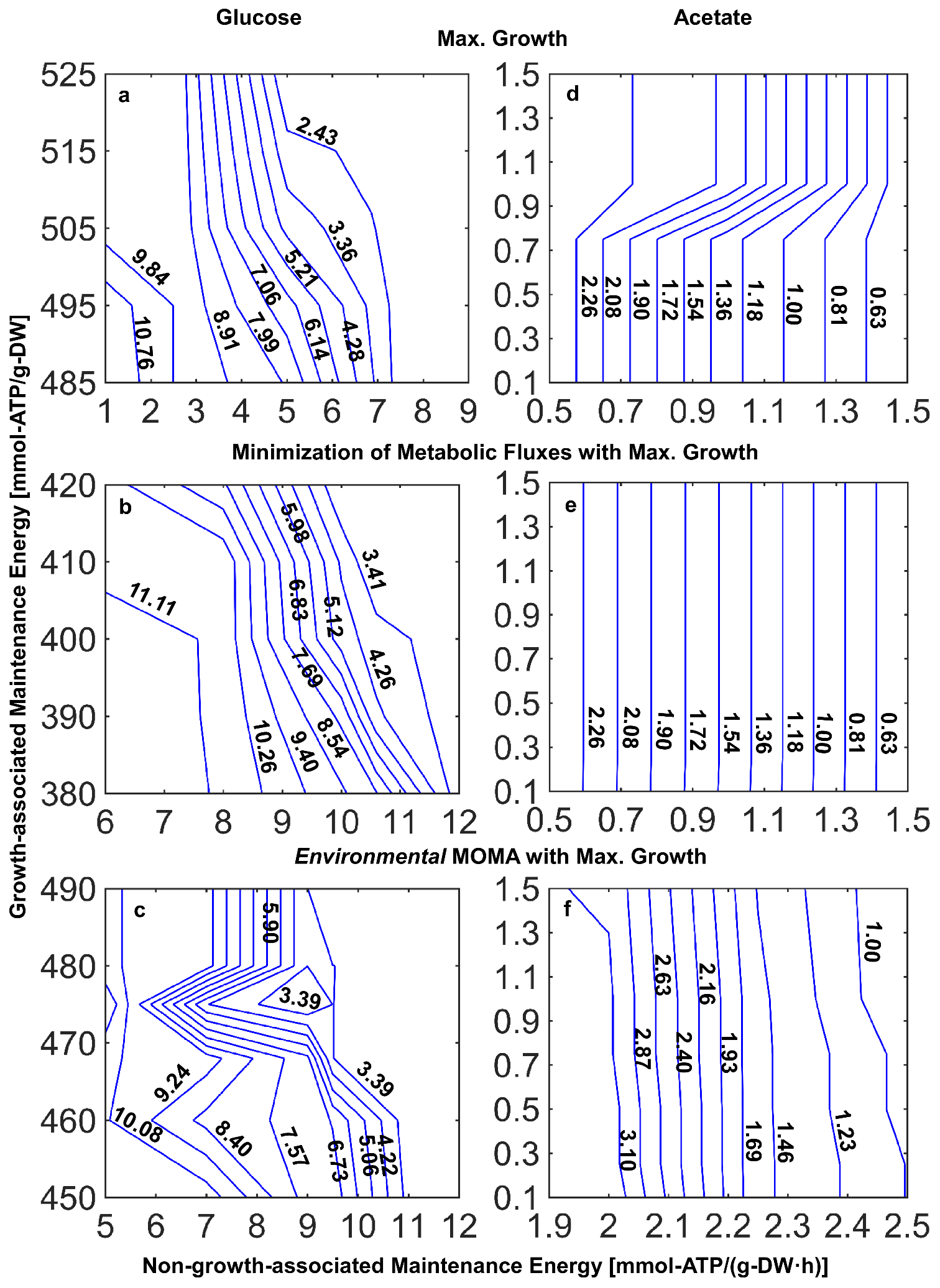
**

**Fig. B.** Contours of the total storage flux [g-COD/(g-COD-Biomass·d)] as a function of the growth- and non-growth- associated maintenance energy (GAM and NGAM, respectively) for the simulated feast growth with glucose (a, b, and c) or acetate (d, e, and f) as carbon substrates. Objective functions used for FBA were maximization of the growth rate alone (maxGrowth, a and d), or in conjunction with minimization of fluxes (minFluxes, b and e) or *environmental* MOMA (c and f).


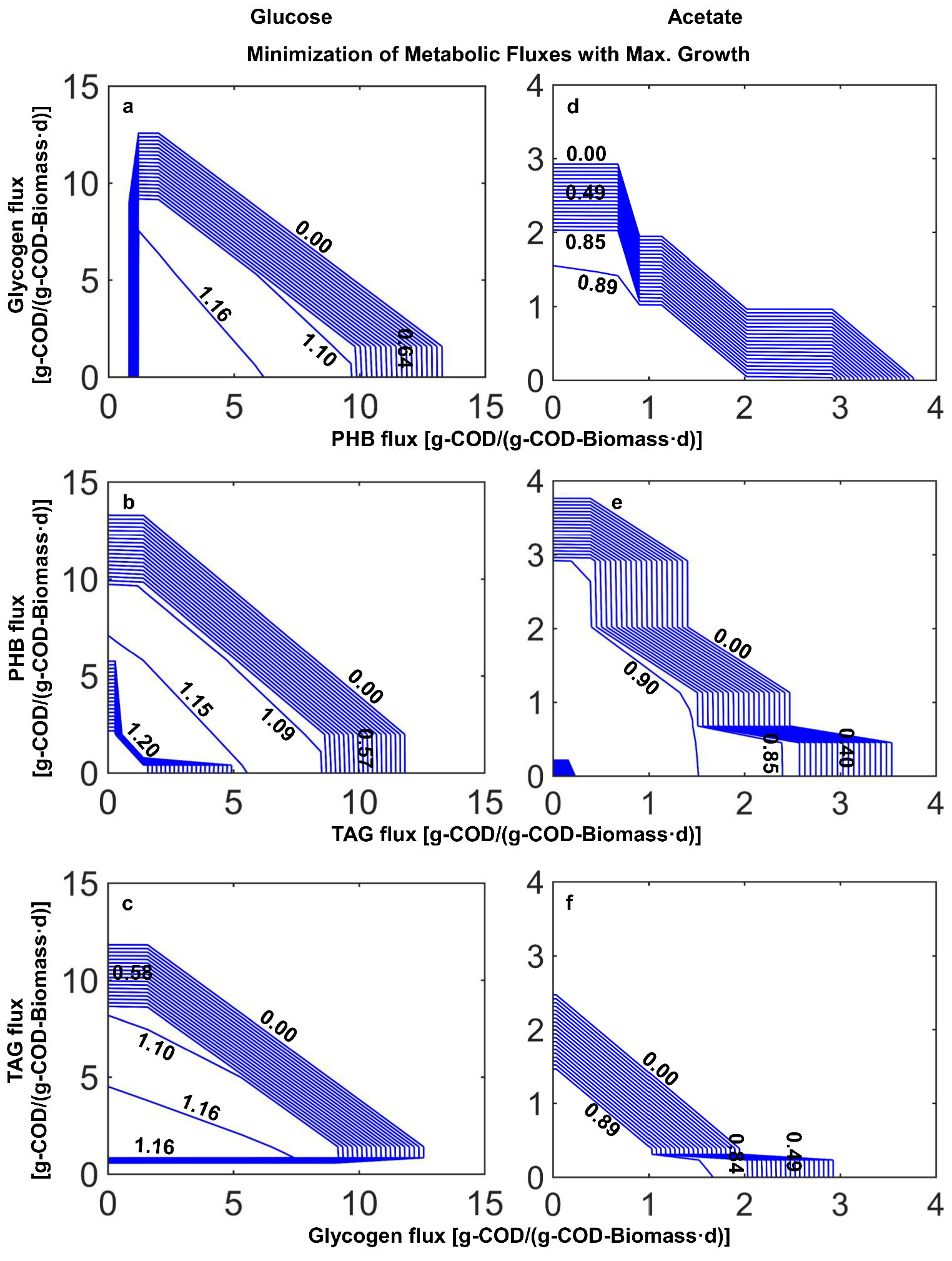


**Fig. C.** Contour plots of flux balance results obtained with the minimization of metabolic fluxes sub-objective function in conjunction with the maximization of growth rate for weighted average maximum growth rates (isolines, units d^−1^) in function of pairs of glycogen and PHB (a and d), PHB and TAG (b and e), and TAG and glycogen (c and f) storage fluxes at experimentally observed glucose - panels a, b, and c: 14.2 g-COD/(g-COD-Biomass·d) - and acetate uptake rates - panels d, e, and f: 4.9 g-COD/(g-COD-Biomass·d). Glycogen fluxes were adjusted to experimentally observed values - panels b and e: 1.3 and 0.03 g-COD/(g-COD-Biomass·d), respectively. PHB fluxes were adjusted to experimentally observed values - panels c and f: 1.6 and 0.9 g-COD/(g-COD-Biomass·d), respectively. TAG fluxes were adjusted to experimentally observed values - panels a and d: 1.1 and 0.3 g-COD/(g-COD-Biomass·d), respectively.

**
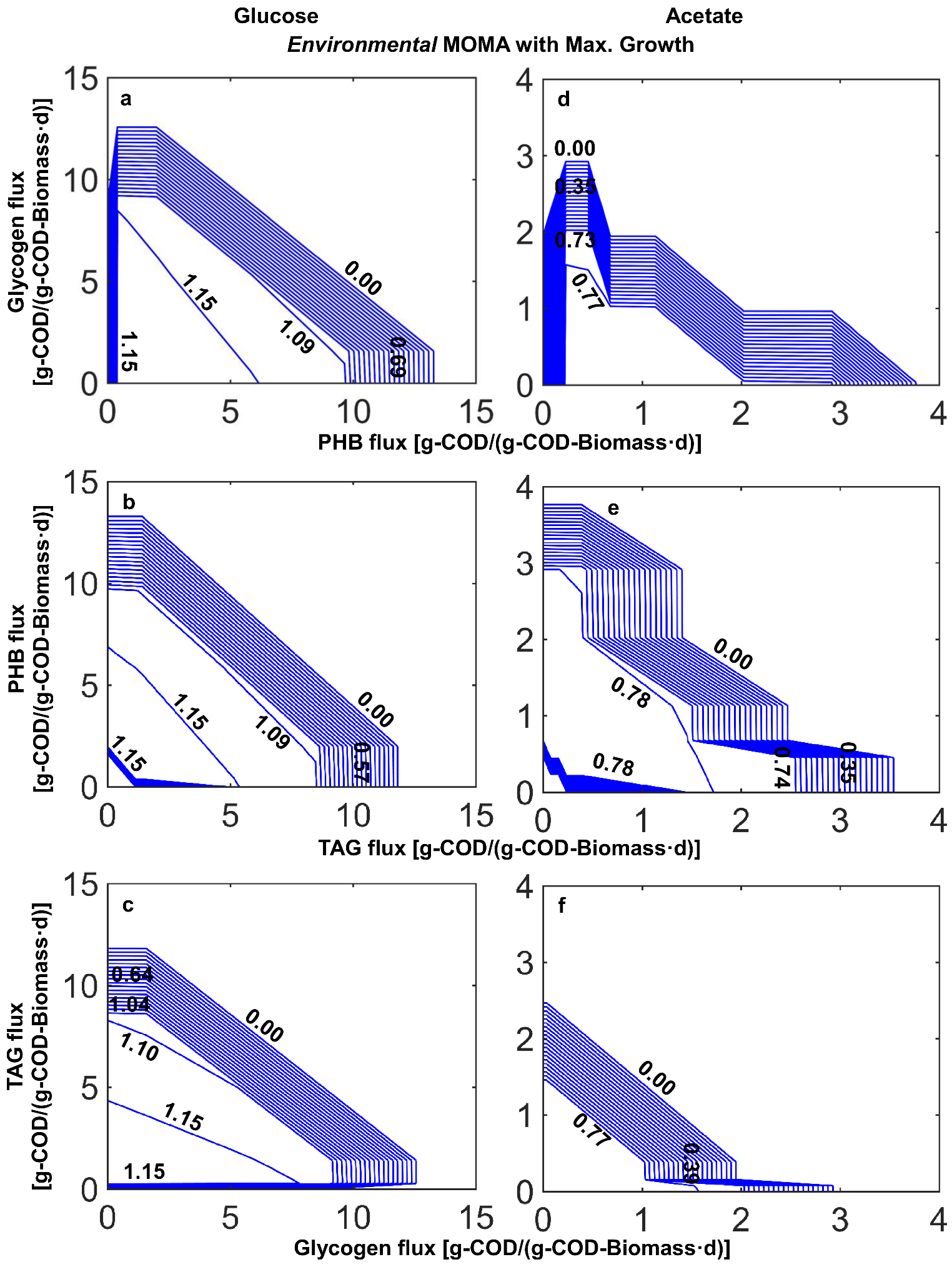
**

**Fig. D.** Contour plots of flux balance results obtained with the *environmental* MOMA sub-objective function in conjunction with the maximization of growth rate for weighted average maximum growth rates (isolines, units d^−1^) in function of pairs of glycogen and PHB (a and d), PHB and TAG (b and e), and TAG and glycogen (c and f) storage fluxes at experimentally observed glucose - panels a, b, and c: 14.2 g-COD/(g-COD-Biomass·d) - and acetate uptake rates - panels d, e, and f: 4.9 g-COD/(g-COD-Biomass·d). Glycogen fluxes were adjusted to experimentally observed values - panels b and e: 1.3 and 0.03 g-COD/(g-COD-Biomass·d), respectively. PHB fluxes were adjusted to experimentally observed values - panels c and f: 1.6 and 0.9 g-COD/(g-COD-Biomass·d), respectively. TAG fluxes were adjusted to experimentally observed values - panels a and d: 1.1 and 0.3 g-COD/(g-COD-Biomass·d), respectively.
